# Improvement and Innovation of Cryopreservation and In Vitro Methods in Plant Resource Protection

**DOI:** 10.3390/biology13090741

**Published:** 2024-09-21

**Authors:** Haeng-Hoon Kim, Elena Popova

**Affiliations:** 1 Department of Agricultural Life Science, Sunchon National University, Suncheon 57922, Republic of Korea; 2 K.A. Timiryazev Institute of Plant Physiology, Russian Academy of Sciences, Botanicheskaya 35, Moscow 127276, Russia

Plant genetic resources (PGRs) are perhaps the most precious gift of nature to humanity: they provide food, shelter, medicines, and many goods of high economic value, not to mention their key importance for healthy ecosystems and their aesthetic value. Nonetheless, the depletion of plant biodiversity is a problem that has escalated to a threatening level in recent decades. There is an urgent need for the scientific community to search for and put into practice modern methods for PGR conservation through principal studies and practical applications of tissue culture and cryobiotechnology [1,2].

Over the past decades, cryopreservation has markedly progressed from laboratory experiments using single-genotype materials to large-scale methodology testing on diverse genebank collections [3,4,5]. This move has uncovered new shortcomings and challenges for the methodology, such as considerable variation in recovery response among genotypes, year-by-year reproducibility, the need for cost reduction, and specific issues related to conserving plant material of tropical or subtropical origin with no inherited mechanisms of cold or desiccation tolerance. From the perspective of protocol development, the big challenges lie in the conservation and propagation of endangered species, including aquatic and wetland plants that are hyper-sensitive to desiccation [6,7,8,9], as well as elite genotypes of novel crops of increasing economic value [10,11,12]. These tendencies were reflected in the contributions to the Special Issue, which focused on different aspects of in vitro culture and cryobiotechnology to approach the problem of PGR conservation, including crops, ornamental, medicinal, and model plants, as well as wild species and crop wild relatives.

Kaviani and Kulus [13] reviewed the application of cryobiotechnology to endangered ornamental plants and fruit crops from the tropics and subtropics. These species do not possess natural mechanisms of freezing tolerance and are often very sensitive to dehydration. This complicates the implementation of classical cryopreservation protocols for such species, making their cryobanking challenging and time-consuming. According to the authors, encapsulation-dehydration and encapsulation-vitrification are the most utilized methods for cryopreserving tropical ornamental plants, such as orchids, with shoot tips, protocorms, and PLBs being the most frequently utilized materials. Tropical fruit trees are cryopreserved mostly by vitrification, droplet-vitrification, and D- and V-cryoplate techniques. Here, the material types vary from shoot tips to zygotic embryos, somatic embryos, and embryogenic cell cultures [13]. Not surprisingly, a threshold recovery of 40% is not always possible to achieve with tropical germplasm. Yet, even 20–30% explant recovery to whole plants is a significant step towards cryobanking these challenging but highly economically important crops. The authors also advocate the use of “-omics” technologies (genomics, proteomics, and metabolomics) to assist both protocol optimization and the assessment of the genetic integrity of the recovered plants.

Popova et al. [14] review draws attention to the critical role of regrowth conditions in the post-cryopreservation behavior of in vitro plant germplasm. The authors compiled the data from over 260 experimental and review papers to explore the parallels and differences in optimum post-cryopreservation conditions for a wide range of plant materials, including apical shoot tips, axillary buds, embryogenic and non-embryogenic cell cultures, somatic embryos, hairy and adventitious roots, adventitious buds, rhizome sections, and microtubers, amongst others. The review highlights and discusses major strategies, including the balance of plant growth regulators; medium composition, physical state, and osmotic potential; exogenous antioxidants; polymers; nanoparticles; antimicrobial agents; illumination quality and intensity. These strategies help plant materials combat non-lethal injuries induced by cryopreservation stress. The authors demonstrate how different strategies and their combinations at the post-cryopreservation step support plant material’s rapid recovery and regeneration and advocated for step-wise recovery to improve normal regeneration in stress-sensitive genotypes.

Yuorieva et al. [15] give a birds-eye view of the in vitro and cryobank collections of plant cells and tissues, microalgae, and cyanobacteria at the research institute of the Russian Academy of Sciences. This story is an excellent example of how a research institute benefits from hosting, supporting, and encouraging the collaboration of multiple collections of national importance. The history and current holdings of the collections and their contribution to the institute’s research activities over the years are covered, emphasizing material acquisition strategies, the development of conservation methods, and quality management systems. The review features one of the world’s oldest cryobanks (established in the 1970s) of plant material and collections of biotechnologically important strains of plant cells and adventitious root cultures, microalgae, and cyanobacteria capable of producing bioactive compounds that are beneficial for human health. These strains are directly used in the institute’s biotechnological R&D center, which strongly focuses on collection development and rationalization.

Genetic collections are also in the spotlight in research papers on this Special Issue. Ex situ conservation of fruit trees and strawberries using cryopreservation was explored in the national genebanks of Germany [16], Russia [17], and the Republic of Korea [18]. Höfer and Flachowsky [16], of the Dresden-Pillnitz Fruit Genebank at the Institute for Breeding Research on Fruit Crops in Germany, applied direct dormant bud cryopreservation and the cryopreservation of shoot tips from in vitro culture for its diverse genetic collections of apples and pears [16]. A total of 180 accessions of different *Malus* wild species were cryopreserved using dormant buds over 10 years; 116 (64%) had a viability of over 40%. In *Pyrus* wild species, in vitro shoot tip cryopreservation using the PVS2-based vitrification gave better results than dormant bud cryopreservation in preliminary studies using 35 samples belonging to 21 species [16].

Multi-year experiments performed by Verzhuk et al. [17] in the N.I. Vavilov All-Russian Institute of Plant Genetic Resources (VIR) confirmed that cold storage and cryopreservation were effective for conserving bird cherry (*Padus* Mill.) dormant buds, resulting in over 43% recovery, assessed under both laboratory and field conditions for all five genotypes tested. Moreover, cryopreservation did not affect the total sugar and ascorbic acid content of fruits produced on regenerated trees.

Bae et al. [18] assessed the morphological traits, fruit characteristics, and genetic stability of six strawberry genotypes grown in the greenhouse from cryopreserved shoot tips. They found that cryopreservation induced no abnormalities in plant and fruit characteristics or genomes. However, some variability in sugar content and the pH of fruits in three accessions was observed, it was only in the first runner generation.

Tissue culture is a helpful tool for both the conservation of PGR and the rapid production of improved genotypes, particularly for species with increasing economic value; this was also reflected in two research papers that focused solely on in vitro techniques. Using optimized media, tissue culture was effective in propagating a rare and critically endangered aquatic carnivorous plant, *Aldrovanda vesiculosa*, by Parzymies et al. [19]. Hesami et al. [20] explored phenotypic changes of in vitro-grown *Cannabis* plantlets derived from different types of explants over multiple subculture circles. They confirmed the effectiveness of tissue culture in the propagation of this crop.

In conclusion, we would like to thank all the authors for their papers submitted to this Special Issue, and acknowledge all the reviewers for their thoughtful and helpful comments and time spent with the submissions. We would also like to express our sincere gratitude to the staff of the *Biology* editorial office for their constant and highly professional support.
